# Effect of porosity enhancing agents on the electrochemical performance of high-energy ultracapacitor electrodes derived from peanut shell waste

**DOI:** 10.1038/s41598-019-50189-x

**Published:** 2019-09-20

**Authors:** N. F. Sylla, N. M. Ndiaye, B. D. Ngom, D. Momodu, M. J. Madito, B. K. Mutuma, N. Manyala

**Affiliations:** 1 Department of Physics, Institute of Applied Materials, SARChI Chair in Carbon Technology and Materials, University of Pretoria, Pretoria, 0028 South Africa; 2 Laboratoire de Photonique Quantique Energie, et Nano-Fabrication, Faculté des Sciences et Techniques Université Cheikh Anta Diop de Dakar (UCAD), B.P. 5005 Dakar-Fann Dakar, Sénégal

**Keywords:** Atmospheric chemistry, Supercapacitors

## Abstract

In this study, the synthesis of porous activated carbon nanostructures from peanut (Arachis hypogea) shell waste (PSW) was described using different porosity enhancing agents (PEA) at various mass concentrations via a two-step process. The textural properties obtained were depicted with relatively high specific surface area values of 1457 m^2^ g^−1^, 1625 m^2^ g^−1^ and 2547 m^2^ g^−1^ for KHCO_3,_ K_2_CO_3_ and KOH respectively at a mass concentration of 1 to 4 which were complemented by the presence of a blend of micropores, mesopores and macropores. The structural analyses confirmed the successful transformation of the carbon-containing waste into an amorphous and disordered carbonaceous material. The electrochemical performance of the material electrodes was tested in a 2.5 M KNO_3_ aqueous electrolyte depicted its ability to operate reversibly in both negative and positive potential ranges of 0.90 V. The activated carbon obtained from the carbonized CPSW:PEA with a mass ratio of 1:4 yielded the best electrode performance for all featured PEAs. The porous carbons obtained using KOH activation displayed a higher specific capacitance and the lower equivalent series resistance as compared to others. The remarkable performance further corroborated the findings linked to the textural and structural properties of the material. The assembled device operated in a neutral electrolyte (2.5 M KNO_3_) at a cell potential of 1.80 V, yielded a ca. 224.3 F g^−1^ specific capacitance at a specific current of 1 A g^−1^ with a corresponding specific energy of 25.2 Wh kg^−1^ and 0.9 kW kg^−1^ of specific power. This device energy was retained at 17.7 Wh kg^−1^ when the specific current was quadrupled signifying an excellent supercapacitive retention with a corresponding specific power of 3.6 kW kg^−1^. These results suggested that peanut shell waste derived activated carbons are promising candidates for high-performance supercapacitors.

## Introduction

The global demand for energy increases exponentially due to the new development and skyrocketing consumption of electronic gadgets such as mobile phones, laptops, and hybrid electric vehicles^1^. Furthermore, most of the energy generation sources used are fossil fuel-based (non-renewable) which creates enormous environmental damage. Thus, there is a need to design and fabricate efficient and also environmentally friendly energy storage systems to meet the continuous energy demand. Energy storage technology plays a key role in continuous energy production systems and has a potential to offset the intermittent problem related to some renewable energy sources (solar, wind, wave, etc.)^2^. Actually, batteries and supercapacitors are the most common devices for electrical energy storage^3^. Batteries are currently the primary energy storage system, despite their limitation in safety and operating in high specific power applications. Supercapacitors (SCs), sometimes referred to as ultra-capacitors (or electrochemical capacitors (ECs)) possess high power delivery capability but are plagued with a lower specific energy as compared to batteries^4^. SCs have attracted significant attention due to their long cycle life, stability, low maintenance and efficiency^5,6^. According to their charge storage mechanism, two types of SCs can be classified namely electrical double layer capacitors (EDLCs) and faradic capacitors (FCs). EDLCs store charges electrostatically with the charged ion located at the surface of the electrode material. However, the charge storage of FCs (or pseudocapacitors) involves a fast redox reaction at the electrode-electrolyte interface^7,8^.

Carbon-based materials such as carbon nanotubes (CNTs), activated carbon (AC), carbon aerogels, graphene etc. have been mostly used as the electrodes materials for EDLC due to their high specific surface area, good electrical conductivity and large pore size distribution (micro, meso and macropores)^9,10^.

More importantly, the ACs obtained from biomass sources have attained considerable attention due to their high abundance, relatively low-cost synthesis route and environmentally safe trait. A variety of biomass materials such as oil palm kernel shell^11^, cumin waste^12^, chitosan^13^, cattail^14^, banana peels^15^, orange peels^16^, rice husk^17^ etc., have been used as precursors for producing highly porous AC materials via chemical and physical activation processes. Chemical activation involves the impregnation of carbonaceous raw materials with chemical activating reagents such as potassium hydroxide (KOH), zinc chloride (ZnCl_2_), phosphoric acid (H_3_PO_4_) with subsequent pyrolysis^18,19^. The porosity enhancing agents (PEAs) play an important role in the activation process by creating a porous network, which aids the development of a high specific surface area (SSA). This can contribute to the charge storing ability and also facilitate easy access of the electrolytes ions into the pores of the AC electrode materials.

In the literature, different porosity enhancing agents (PEAs) have been used to produce hierarchically porous carbon materials derived from biomass precursors. For example, M. Sivachidambaram *et al*.^20^ prepared activated carbon from Tamarindus indicaus fruit shell using H_3_PO_4_ activating agent and obtained an 846 m^2^ g^−1^ SSA with a total pore volume of 0.60 m^3^ g^−1^. Similarly, Z. Sun *et al*.^21^ activated raw Pteroceltis tatarinowii bark with ZnCl_2_ and KOH. In their report, the KOH-activated raw material showed a higher SSA of 1721 m^2^ g^−1^ and total pore volume of 0.90 cm^3^ g^−1^ as compared to the one activated with ZnCl_2_ (1210 m^2^ g^−1^ and 0.57 cm^3^ g^–1^, respectively).

Recently, J. Han *et al*.^22^ synthesized giant miscanthus, corn stalk, and wheat stalk-derived activated carbons with KOH and recorded specific surface area values of 2212, 2434 and 2327 m^2^ g^−1^ with a pore volume 0.99, 1.22, 1.10 m^3^ g^−1^ respectively. Generally, most chemical activation studies which reported high porosity proposed potassium-based salts to be more effective in pore creation and activation of carbonaceous materials as compared to other PEAs (H_3_PO_4_, ZnCl_2_, NaOH…)^19,23–25^.

Peanut shell waste is of great interest as it is a common agricultural waste that is cheap, readily available, and can be made useful as a renewable energy source^26^. Recently, activated carbon from peanut shell waste has been generated using ZnCl_2_, FeCl_3_ and H_3_PO_4_ and applied in supercapacitors, Lithium and Sodium batteries and as well as in wastewater treatment^27–30^. These results on peanut shell waste derived activated carbons suggested an improvement in the pore creation during carbonization/activation process due to the multiple composition of the peanut shell such as lignin, cellulose and hemicellulose^31^. However, a study of the effect of potassium based porosity enhancing agents on the morphological, structural and electrochemical properties of peanut shell waste derived carbons to our knowledge has not been explored.

In this work, we therefore focus our study on the synthesis of hierarchically porous activated carbon from peanut (Arachis hypogea) shell waste (PSW) via two steps (carbonization/activation) using different potassium-containing PEAs namely KHCO_3_, K_2_CO_3_ and KOH with varying mass concentrations. In addition, the effect of the various mass concentrations on the textural and electrochemical properties of the activated carbon-based material electrodes for supercapacitor devices was explored. The PSW activated with KOH at a ratio of 1:4 (APSW-KOH4) exhibited the best initial electrochemical performance in half-cell configuration in 2.5 M KNO_3_. The choice of this neutral electrolyte was based on the pH value which controls the ability to operate in a wider potential window due to the electrolyte stable potential windows (ESPWs) as compared to acidic or alkaline aqueous electrolytes which is limited by the hydrogen evolution reaction (HER) and oxygen evolution reaction (OER)^32–34^.

Additionally the size of the ions in the KNO_3_ (K^+^, NO_3_^−^) ions are suitable to be transported within the pores of the as-synthesized APSW sample. The best charge transport dynamics was recorded in the APSW-KOH4 sample which gives the ideal pore sizes for efficient ion transport and charge storage. KNO_3_ aqueous electrolyte also has a high conductivity, is environmentally friendly, non-corrosive and cheap to synthesize^35,36^. Thus, symmetric cells composed of APSW-KOH4//APSW-KOH4 electrodes material were assembled and tested. The full device displayed a high specific energy of 25.2 Wh kg^−1^ with a corresponding high specific power of 0.9 kW kg^−1^. A good device stability was demonstrated with an 83% capacitance retention up to 15,000 cycles. Amazingly, the device further showed an enhancement in the performance after being subjected to voltage holding (floating) tests for more than 6 days (150 h) floating time.

## Experimental Method

### Material synthesis

The porous activated carbon nanostructures were obtained via a two-step pyrolysis technique of peanut shell waste (PSW) material at elevated temperatures. The collected PSW was initially washed with deionized water (DI) thoroughly and dried over 12 h under direct sunlight. Then, the dried PSW was ground into fine powder. Thereafter, 5 g of PSW powder was mixed with 0.5 g of urea, 1 g of NaCl and a few drops of deionized water (DI) to make a sludge. A compact rectangular block (B-1) from the sludge mixture was placed in an oven at 80 °C to dry overnight.

The dried compact block mixture was transferred into a horizontal tube furnace set at 600 °C (ramp rate of 5 °C min^−1^) for a period of 2 h in 250 sccm flow of Argon gas. The black product obtained (referred to as carbonised peanut shell waste (CPSW)) was washed severally with DI and dried at 80 °C.

For the activation process at elevated temperature, a weighed amount of CPSW was mixed with three different porosity enhancing agents (PEAs) namely, KHCO_3_, K_2_CO_3_ and KOH, at different mass loading ratios of 1:1, 1:2, 1:4 and 1:6 for the CPSW:PEA, respectively. The moulded mixtures in form of blocks of CPSW:PEA at different ratios were also prepared to ensure an efficient contact between the PEA and the material surface. The moulded blocks (B-2) were subjected to pyrolysis at 850 °C using a 5 °C min^−1^ ramp rate for a 1 h period using 250 sccm of Argon gas flow. The resulting products were soaked in 3 M HCl and sonicated for 8 h to get rid of the excess unreacted PEA. Finally, the filtered solids materials were rinsed with DI until a neutral pH before drying at 80 °C for 12 h. The activated carbon nanostructures from peanut shell waste are indicated as APSW-Yx where Y denotes the PEA and x the mass ratio of PEA.

### Materials characterization

The material textural features were determined by using the Brunauer-Emmett-Teller (BET) technique measured from a Micrometrics TriStar II 3020 (version 2.00) system at −196 °C. Furthermore, porosity properties i.e. the pore size distribution (PSD) was analysed with the Barrett-Joyner-Halenda (BJH) and density functional theory (DFT) plots. The Raman spectra of the APSW material was performed using a WITec confocal Raman microscope (WITec alpha300 RAS+) operating with a 532 nm laser set at a 5 mW emitting power to minimize heating effects.

X-ray photoelectron spectroscopy (XPS) analysis of the synthesized material was done using a Thermo Fisher photoelectron spectrometer fitted with a monochromatic Al Kα radiation X-ray source. A Zeiss Ultra-plus 55 field emission scanning electron microscope (FE-SEM), operated at 1.0 kV was used to characterize the surface morphologies of the samples. TEM measurements were obtained using a cold field-emission JEOL F200 TEM operating at 200 kV.

### Electrochemical characterization

The electrochemical measurement in the three and two electrodes was performed using a Biologic VMP-300 16-channel potentiostat (Knoxville, USA). The preparation of the electrode was done by mixing 80% of the active material (APSW-Yx), 10% polyvinylidene difluoride (PVDF) and 10% carbon acetylene black (CB). Thereafter, few drops of N-methylpyrrolidone (NMP) were added to the mixture to make a slurry in an agate mortar. This mixture was pasted onto nickel foam-graphene (NFG) current collector and then dried in an electric oven for 12 h at 60 °C to ensure complete evaporation of the NMP. The three-electrode analysis (half-cell test) was done using a glassy carbon counter electrode (CE) and Ag/AgCl (in saturated KCl) reference electrode (RE). The symmetric device was assembled in a Swagelok system with a glass microfiber filter paper to separate the two electrodes (full-cell). The mass per area of the single electrode in the cell was approximately 3 mg cm^−2^.

All electrochemical test such as cyclic voltammetry (CV), galvanostatic charge-discharge (GCD), electrochemical impedance spectroscopy (EIS) and stability tests were carried out with the APSW-Yx materials operated in a 2.5 M KNO _3_ aqueous electrolyte.

## Results and Discussion

### Textural, structural and morphological characterization

**Figure 1:**
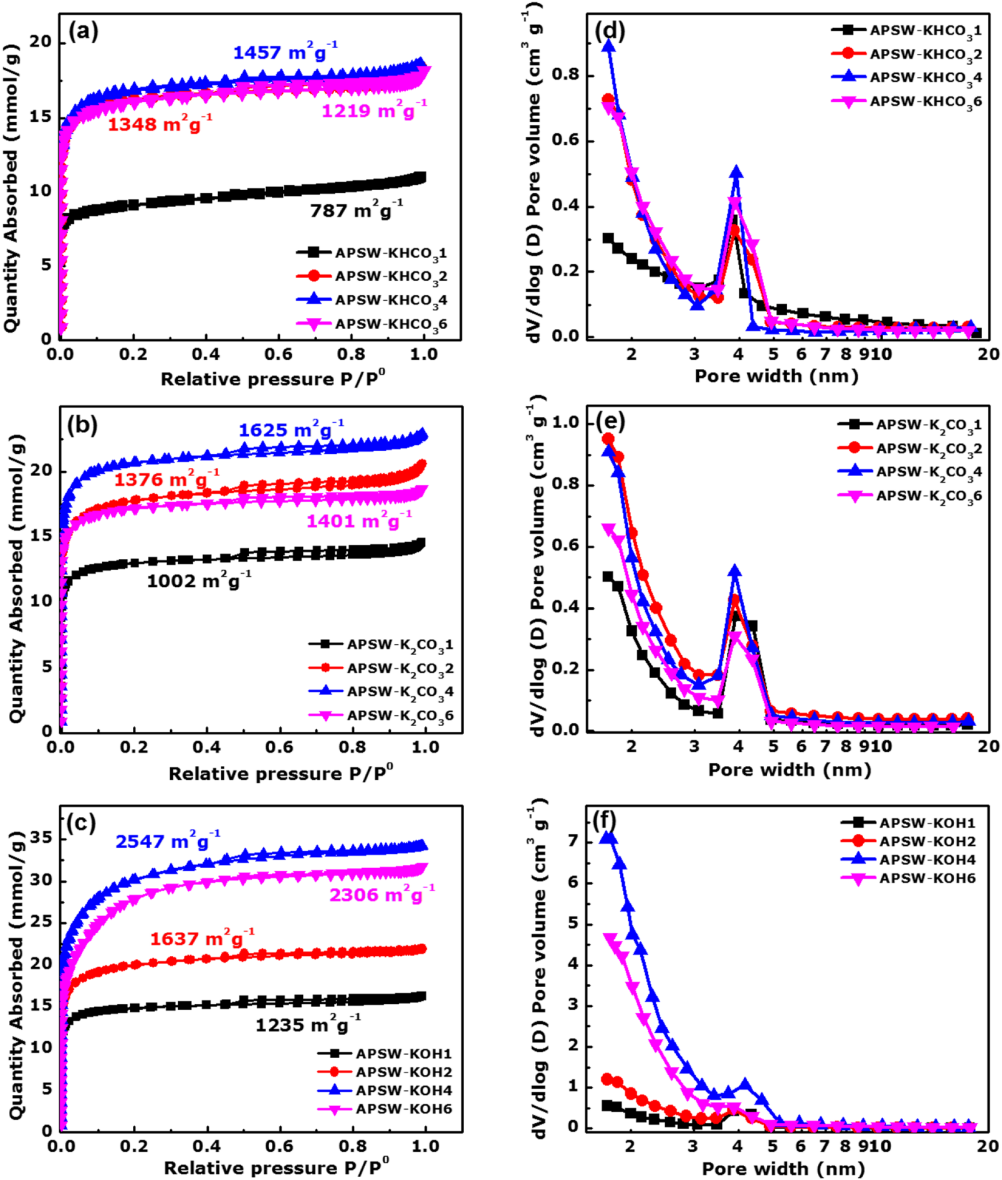
(**a**–**c**) N_2_ absorption-desorption isotherms; (**d**–**f**) pore size distribution data of the activated carbon from peanut shell waste for varying porosity enhancement agents KHCO_3_, K_2_CO_3_ and KOH contents respectively.

The N_2_-adsorption/desorption analysis was used to determine the textural properties of the APSW with different PEA as shown in Fig. 1.

Figure 1(a–c) displays isotherms with indicated BET SSA for the APSW for each PEA (KHCO_3_, K_2_CO_3_ and KOH) with different mass ratios (1:1, 1:2, 1:4 and 1:6). The adsorption isotherms for all the APSW-Yx exhibit a hybrid of type I and IV isotherms with a H4 hysteresis loop according to the IUPAC. At low relative pressure (*P*/*P*_0_ < 0.01) the adsorption isotherm shows a steep uptake of N_2_ which is associated to a well-developed micropores. An hysteresis loop between the adsorption and desorption branches indicates the presence of mesoporous at a medium pressure, while a little adsorption increase was observed relating to the existence of macropores at high pressure (*P*/*P*_0_ > 0.9)^37,38^. In the samples, APSW-KHCO_3_4, APSW-K_2_CO_3_4 and APSW-KOH4 exhibited rises of isotherm at P/P_0_ of 0.43, and a small hysteresis loop occurs at P/P_0_ of 0.43–0.70. The appearance of the hysteresis loops indicate the existence of a bit bigger pore^39^. It can been seen that the APSW-Yx reveal a hierarchically porous texture with a coexistence of micro, meso and macropores which provide fast interfacial ion-transport kinetics and efficient ion accessible surface area for charge storage^40,41^.

Figure 1(d–f) shows the Barrett-Joyner-Halenda (BJH), which determined the pore size distribution (PSD) analysis of the APSW-Yx samples. The PSD observed peaks confirm the presence of micropores and mesopores. However, the presence of macropores is not seen in the PSD plots due to limitation in the pore width range displayed. The SSA and the total pore volume of the APSW-Yx are summarized in Fig. S1(a,b). The SSA and the total pore volume kept increasing as the amount of PEA added was increasing up to a threshold (1:4) then experienced a decrease with further increase in PEA (1:6).

The same trend was observed for all porosity enhancing agents used in this study.

The surface areas of all prepared materials are shown in Table S1 therefore APSW-KHCO_3_4, APSW-K_2_CO_3_4 and APSW-KOH4 exhibit the higher SSA 1457 m^2^ g^−1^, 1625 m^2^ g^−1^ and 2547 m^2^ g^−1^, respectively as compared to the others carbon materials. In addition, the sample activated with KOH in a mass ratio of 1:4 displayed the highest SSA of 2547 m^2^ g^−1^ and largest total pore volume of 1.2 cm^3^ g^−1^. The highest SSA and the total pore volume could be attributed to the multiple decomposition steps for the KOH as compared to other PEAs. In the course of the activation with the different PEAs at elevated temperature, the decomposition reaction taking place results in the improvement of the porosity via gasification from the generated carbon dioxide (CO_2_) and carbon monoxide (CO) gas^25^. In addition, the H_2_ gas produced during KOH decomposition could etch the carbon atoms creating more pores^42^. The process of activation with the PEAS (KOH, K_2_CO_3_ and KHCO_3_) within the sample can be described by the following reactions^25,43,44^:

1 $$6{KOH}+2{C}\leftrightarrow 2{K}+3{{H}}_{2}+2{{K}}_{2}{C}{{O}}_{3}$$

2 $${{K}}_{2}{C}{{O}}_{3}\leftrightarrow {{K}}_{2}{O}+{C}{{O}}_{2}$$

3 $${{K}}_{2}{C}{{O}}_{3}+{C}\leftrightarrow {{K}}_{2}{O}+2{CO}$$

4 $${{K}}_{2}{O}+{C}\leftrightarrow 2{K}+{CO}$$

5 $$2{KHC}{{O}}_{3}+2{C}\leftrightarrow {{K}}_{2}{C}{{O}}_{3}+{C}{{O}}_{2}+{{H}}_{2}{O}$$

During this process, the KOH reacts with the carbon (C) to generate K_2_CO_3_ and metallic potassium as shown in Eq. (1) at 400 °C. At higher temperatures (>700 °C), the K_2_CO_3_ decomposes to give potassium oxide (K_2_O) and CO_2_ (Eq. 2). Additionally, the K_2_CO_3_ could react with the C to yield K_2_O and CO thus, creating pores within the carbon matrix (Eq. 3). Finally, the generated K_2_O undergoes further reaction with C to give K and CO. The K intercalates into the carbon matrix and be readily removed by washing the final products resulting in the creation of irregular channels and pores within the APSW (Eq. 4). Similarly, the KHCO_3_ undergoes initial decomposition to give K_2_CO_3_ as shown in Eq. 5^25^. Consequently, the K_2_CO_3_ further reacts with carbon or decomposes to give CO and CO_2_ emission as shown previously in Eqs 2–4.

The variation of activating agents was done in order to select the best sample for the electrochemical investigation of electrode device. For example, the high specific surface area and larger of porous carbon material could give better electrochemical capacitance^39,45^.

**Figure 2:**
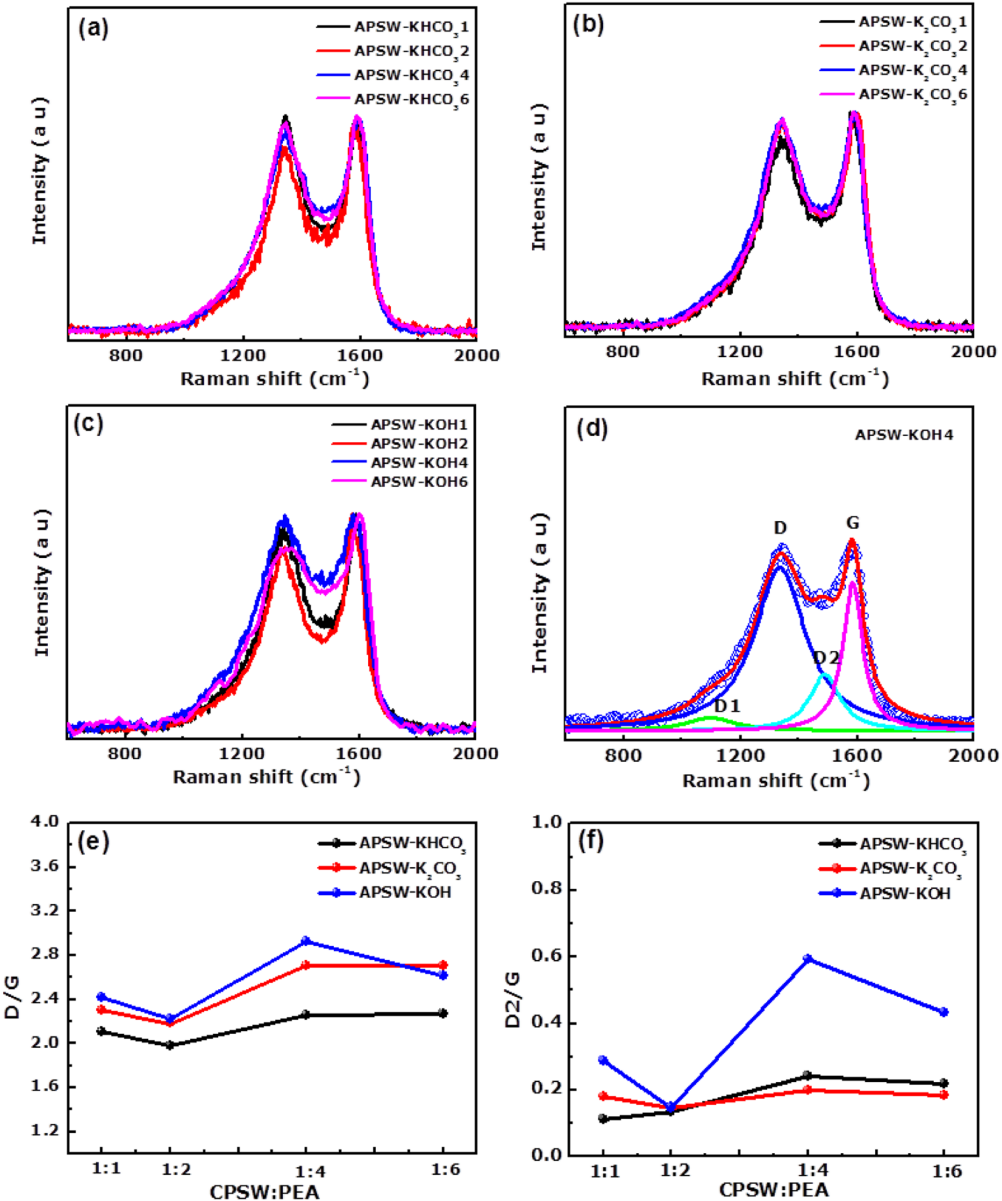
Raman spectra of the APSW samples at different mass ratio with: (**a**) KHCO_3_, (**b**) K_2_CO_3_ and (**c**) KOH PEA respectively, (**d**) deconvolution of the Raman spectrum for APSW-KOH4 sample, (**e**) D/G and (**f**) D2/G ratios as function of APSW-KHCO_3_, APSW-K_2_CO_3_ and APSW-KOH at different mass ratios.

The Raman spectra of the activated peanut shell waste samples were used to investigate the combined effect of the carbonization and activation at elevated temperature processes on the structure of the samples. Figure 2 shows the Raman spectra of the samples for three different PEAs (KHCO_3_, K_2_CO_3_ and KOH). In all Figures, the Raman spectra depict the characteristic D (~1340 cm^−1^) and G (~1583 cm^−1^) bands attributed to carbonaceous materials with A_1g_ and E_2G_ symmetry. The D band arises due to defects or disorder in the graphitic structure of the carbon material and the G band is a characteristic feature of the graphitic material and corresponds to the tangential vibration of the sp^2^ carbon atoms^46^.

In Fig. 2(a–c), the Raman spectra for the APSW-KHCO_3_, APSW-K_2_CO_3_ and APSW-KOH samples at different ratios in the range of 1 to 6 are displayed respectively.

The Raman spectra of the samples were deconvoluted using a Lorentzian function (see Fig. S2 in the supporting information) to obtain the integral areas of the D and G peaks which were used to calculate the D/G ratio of the disordered carbon (D) relative to the graphitic carbon (G). Figure 2(d) shows an example of the deconvoluted Raman spectrum for the APSW-KOH4 sample. In Fig. 2(d), the D1 and D2 bands correspond to the vibration of the sp^2^-sp^3^ bonds and amorphous carbon at interstitial sites of the disordered graphitic structure of the carbon material, respectively^46^.

Figure 2(e) displays a plot of (D/G) ratio of the APSW-Yx at different ratios (1 to 6) which reveals a higher ratio of disordered carbon relative to the graphitic carbon for an activating ratio of 1:4 for all PEAs.

The sample APSW-KOH4 (2.9) is slightly higher compared to APSW-KHCO_3_4 (2.3) and APSW-K_2_CO_3_4 (2.7)_._ Similarly, Fig. 2(f) shows the plot of the D2/G ratio as a function of mass concentration for the various PEAs. It was observed that a higher ratio (or concentration) of amorphous carbon (D2) relative to the graphitic carbon (G) is recorded for the activating ratio of 1:4 for all PEAs, showing the values of APSW-KOH4, APSW-KHCO_3_4 and APSW-K_2_CO_3_4 as 0.60, 0.20, 0.18 respectively. Moreover, the effective crystallite size, *L*_a_ was calculated using the Knight formula^47,48^:

6 $${{L}}_{{a}}=\frac{{C}(\lambda )}{D/G}$$

where the D/G represents the ratio of the integrated area of D to G band, and *C* (*λ*) = 4.96 nm. *C* (*λ*) is the wavelength-dependent pre-factor estimated as *C* (*λ*) ≈ *C*_0_ + *λC*_1_ (for 400 < *λ* < 700 nm range in which *λ* = 532 nm for our current study. *C*_0_ and *C*_1_ are estimated to be −12.6 nm and 0.033 nm, respectively, from a plot of *C*(*λ*) as function of frequency (see ref.^47^). APSW-KOH4 sample displays a smaller effective crystallite size compared to other APSW-Yx (see Fig. S3 and Table S2).

Briefly, a higher ratio of disordered carbon relative to the graphitic carbon and the smaller effective crystallites size observed for APSW-KOH4 sample compared to APSW-KHCO_3_4, and APSW-K_2_CO_3_4 samples could be a reason for high SSA and the total pore volume observed for KOH as compared to other PEAs which could be attributed to the multiple decomposition steps of KOH (Eqs 1–5) during activation at elevated temperature.

**Figure 3:**
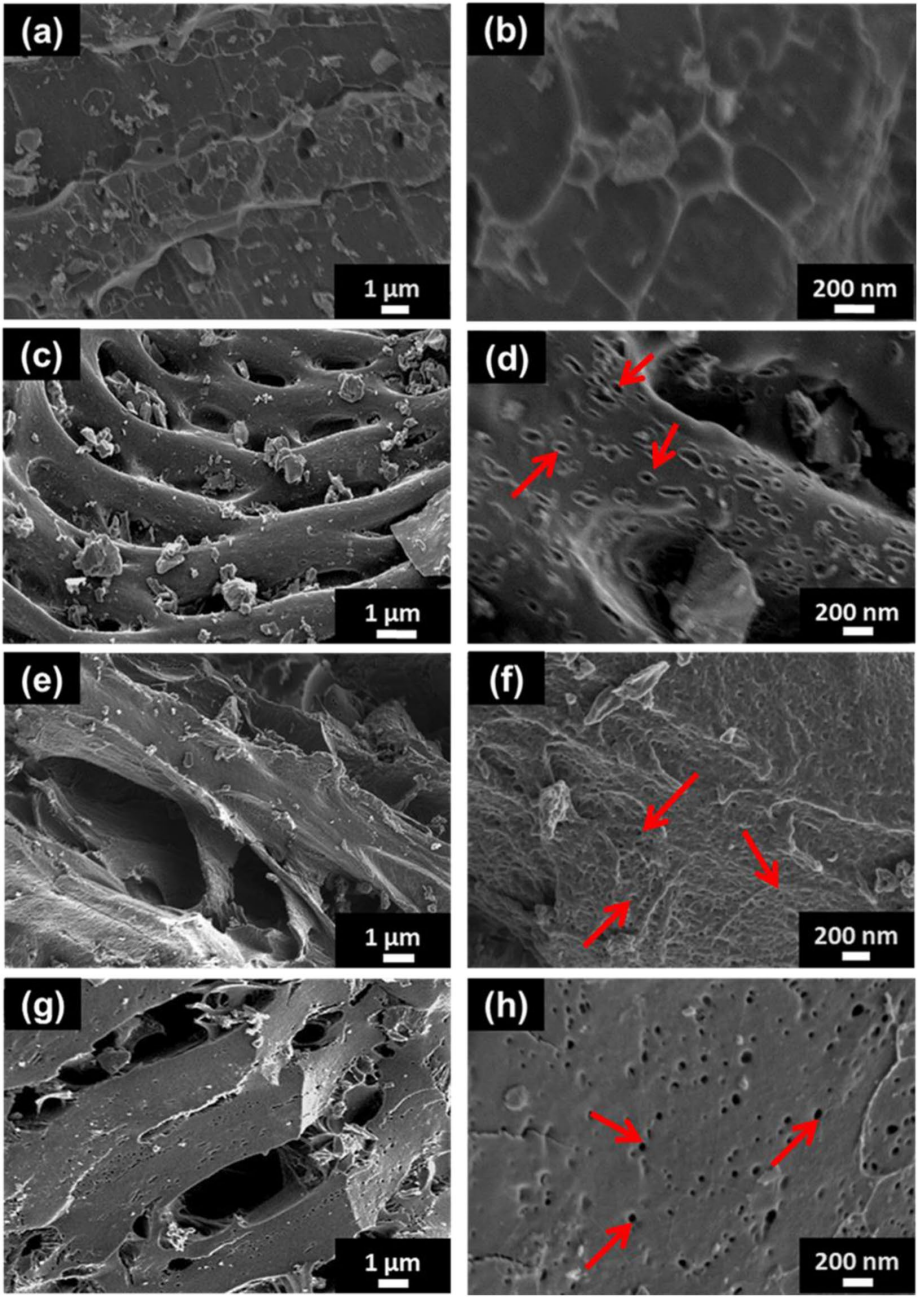
SEM images at low and high magnification of (**a**,**b**) CPSW; (**c**,**d**) APSW-KHCO_3_4; (**e**,**f**) APSW-K_2_CO_3_4 and (**g**,**h**) APSW-KOH4.

Figure 3 shows the scanning electron micrographs of the peanut shell waste samples at low and high magnifications before and after subjection the activation at elevated temperatures. The micrographs of the carbonized sample presented in Fig. 3(a,b) display a sheet-like morphology with a relatively mutilated surface at higher magnification as compared to the raw material (PSW) which shows agglomerated nanoparticles (see Fig. S4).

However, the effect of the activating agents KHCO_3_, K_2_CO_3_, and KOH with a mass ratio of 1:4 leads to the creation of irregular connected microcavities like morphology for all the samples as observed on the morphology on the samples displayed in Fig. 3(c–h). It is noteworthy to state that the micropores and mesopores are not visible from the microscopy analysis. Figures (c), (e) and (f) show the presence of the isolated nanocavities in the samples (illustrated with arrows). It is clear that the morphology of the APSW-KOH4 sample presents larger cavities with a dense distribution of relatively smaller-sized nanocavities on the surface (see high magnification SEM image in Fig. 3(h)) due to the reactivity of KOH^37^. Figure S5 displays the high resolution transmission electron microscopy (HRTEM) micrographs of the carbon materials before and after being activating with a mass ratio of 1 to 4. The HRTEM images confirm the SEM of the as prepared carbon materials. The insets Fig. S5 reveal same morphology as of the activated materials which confirm the SEM analysis. Such channels (microcavities and nanocavities) allow good accessibility of electrolytes into the material allowing good wettability and thereby a better material pores/electrolyte ions interaction^49^.

**Figure 4:**
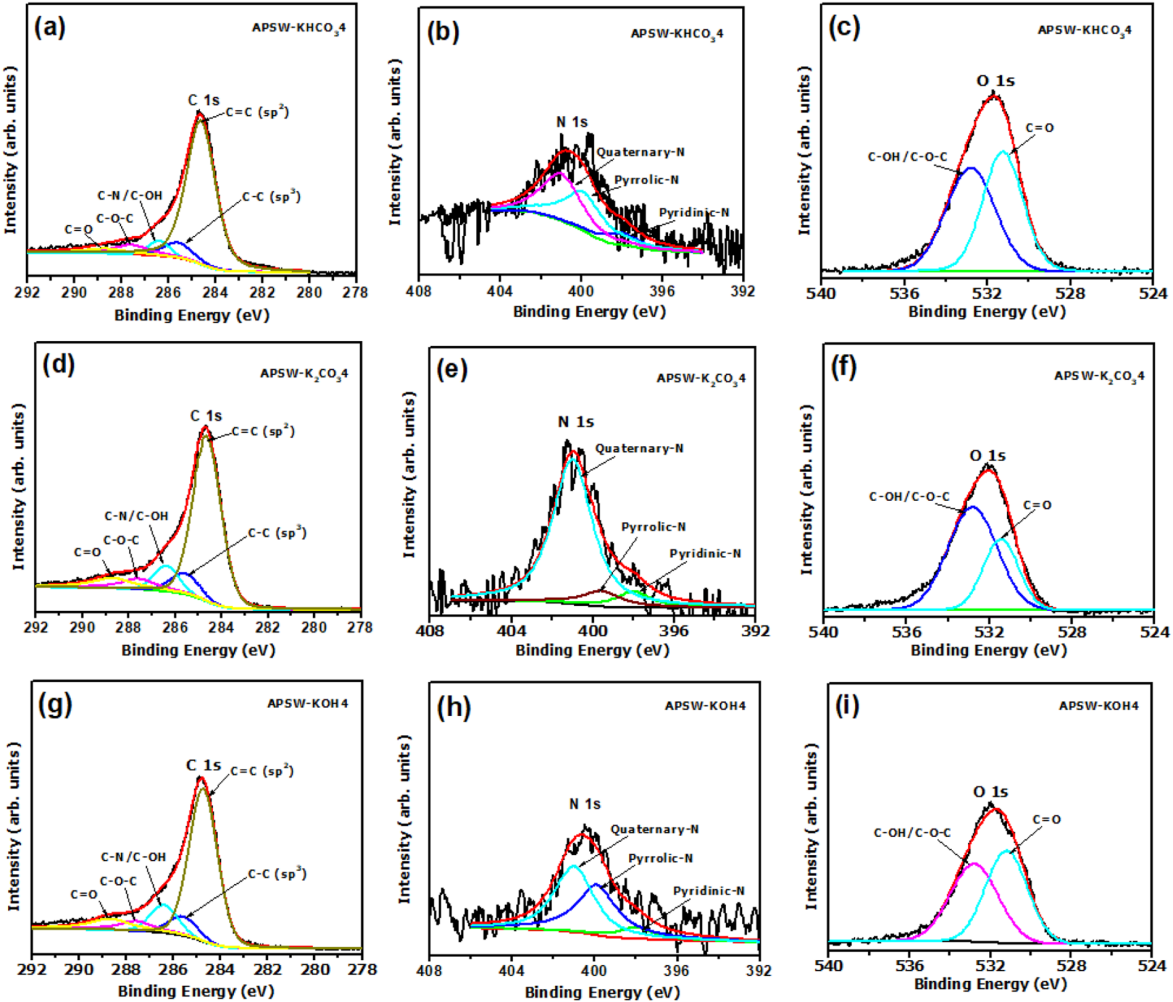
Deconvoluted core levels XPS spectra (C 1 s, N 1 s and O 1 s) of (**a**–**c**) APSW-KHCO_3_4; (**d**–**f**) APSW-K_2_CO_3_4 and (**g**–**i**) APSW-KOH4.

The XPS spectra showing the chemical state of the APSW-Y4 samples as displayed in Fig. 4. To determine the elemental composition (at.%), XPS analysis of all the APSW samples obtained with a mass ratio of 1:4 was performed (Fig. 4). All the samples showed a high carbon content (>85 at.%), an oxygen content of 11–14 at.% and 0.6–1.2 at.% N (see Table S3). The low nitrogen content is proposed to be due to the evolution of nitrogenous volatile products during the carbonization process^50^. The APSW-KHCO_3_4 gave the highest oxygen content agreeing with earlier reports in literature^25^. However, the use of K_2_CO_3_ as a porosity-enhancing agent allowed the retention of a higher nitrogen content within the activated carbon matrix as compared to the other PEAs.

As expected, the APSW-KOH4 with the highest surface porosity gave a low nitrogen content indicating the loss of a higher number of nitrogenous groups during the activation process. Although the nitrogen content for the APSW-KOH4 was low, slightly higher carbon content was present as compared to the APSW-KHCO_3_4 and APSW-K_2_CO_3_4. This suggests that the carbon matrix of the APSW-KOH4 was comprised of more defective carbons in the form of sp^2^/sp^3^ carbon domains and/or sp^3^ bonded nitrogen, which corroborated with the high D/G ratios observed in the Raman measurements.

Figure 4 displays the deconvolution of the high resolution XPS signals of C1S, N1S and O1S in APSW-KHCO_3_4, APSW-K_2_CO_3_4 and APSW-KOH4 materials. Figure 4(a,d,g) show the XPS core level spectra of C 1 s of the APSW-Y4 samples which were deconvoluted into 5 peaks linked to carbon components namely, C = C (sp^2^ carbon, 284.7 eV), C–C (sp^3^ carbon, 285.3 eV), C–OH/C–N (286.5 eV), C–O–C (287.6 eV) and C = O (288.9 eV), respectively^46,47^. The XPS core level spectra of N 1 s of the APSW-Y4 samples (Fig. 4(b,e,h)), reveals three different nitrogen configurations; pyridinic-N (398.0 eV), pyrrolic-N (399.6 eV), and quaternary-N (401.0 eV) which are typically found in N-containing carbon materials^51^. Hence, the prepared and pristine (raw) materials exhibits the same carbon, nitrogen and oxygen components

In aqueous electrolyte-based systems, the pyrrolic-N and pyridinic-N provide hydrophilic polar sites for better charge accumulation on the activated sample surface while the quaternary-N influences carbon conductivity^52,53^.

The O 1 s spectra of the APSW-Y4 samples (Fig. 4(c,f,i)) displayed binding energies at 532.7 and 530.9 eV corresponding to the C–OH/C–O–C and C = O, respectively^54^. The presence of oxygen and nitrogen functional groups could aid in electrode wettability and impact on the electrochemical performance^25,55^.

### Electrochemical characterization

The electrochemical performance of the activated peanut shell waste sample was analysed firstly in three-electrode measurement using 2.5 M KNO_3_ aqueous electrolyte as shown in Figs S4–S6.

**Figure 5:**
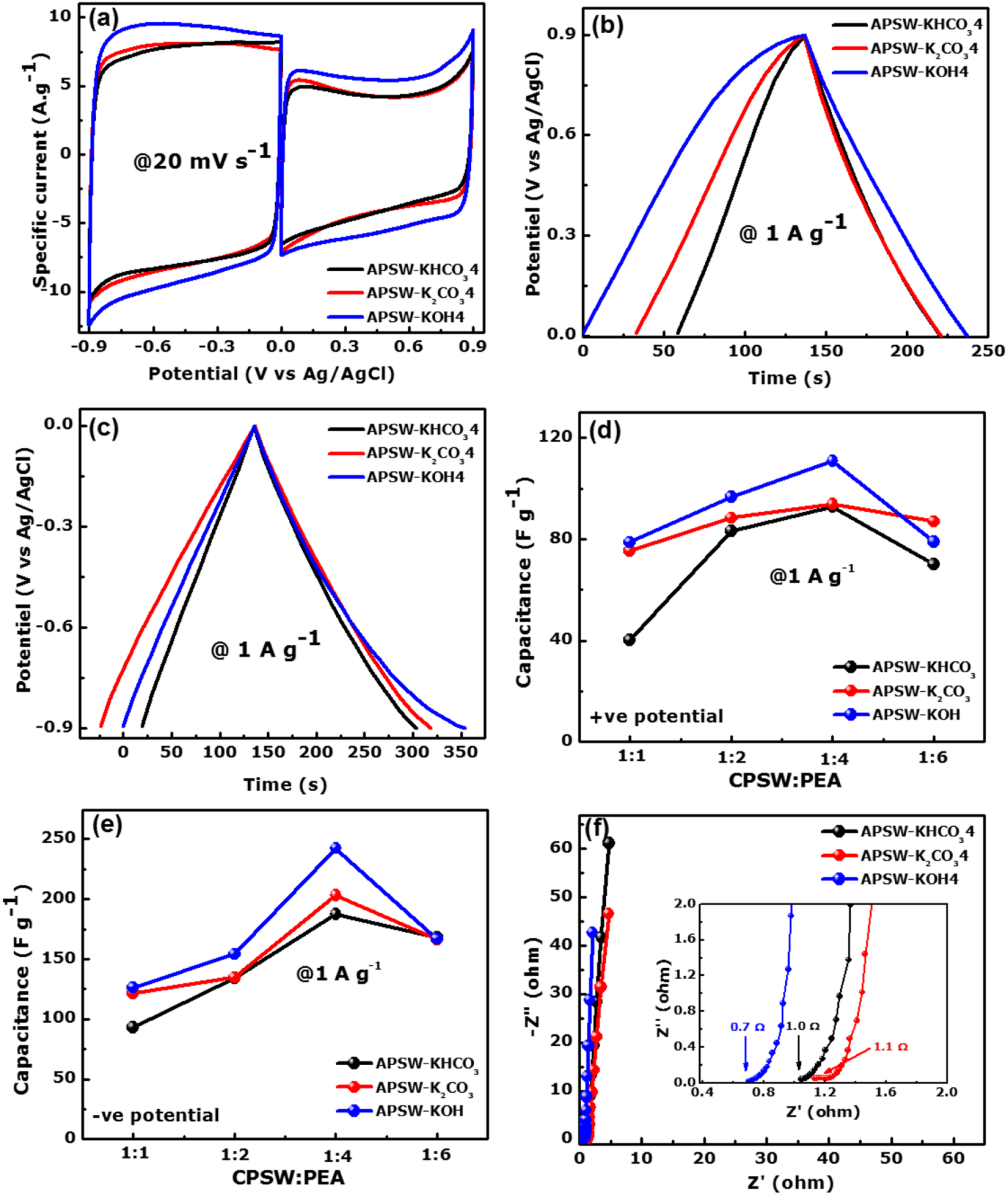
(**a**) CV curves at 20 mV s^−1^ scan rates in positive and negative potential windows, (**b**,**c**) GCD plots at 1 A g^−1^ in positive and negative potential windows, respectively of the APSW-Y4 electrodes, (**d**,**e**) specific capacitance as a function of the ratio CPSW:PEA in positive and negative potential windows, (**f**) Nyquist plot of the APSW-Y4 samples.

From all the electrochemical results presented, it is clear that the materials synthesized with a CPSW:PEA mass ratio of 1:4 gave the highest electrochemical performance. Figure 5(a) displays the cyclic voltammogram (CV) curves at a scan rate of 20 mV s^−1^ of the samples activated with KHCO_3_, K_2_CO_3_ and KOH at mass ratio 1:4.

All the CV plots demonstrated a typical EDLC behaviour with its characteristic rectangular outline in both negative (−0.9–0.0 V) and positive (0.0–0.9 V) potential window ranges. Furthermore, it is seen that the APSW-KOH4 sample exhibits a higher current response in both potentials compared to other APSW-Y4. Figure 5(b,c) present the galvanostatic charge-discharge (GCD) profiles at a specific current of 1 A g^−1^ operating in the same potential window in both negative and positive ranges (0.0–0.9 V and −0.9–0.0 V) as reported for the cyclic voltammetry tests. The GCD curves display a triangular shape with a linear discharge profile indicating a typical capacitive nature. However, activated sample APSW-KOH4 displayed a longer discharge time as compared to the APSW-KHCO_3_4 and APSW-K_2_CO_3_4. The specific capacitance (*C*_S_ in F g^−1^)) of the sample electrodes as a function of the CPSW:PEA is illustrated in Fig. 5(d,e) in the positive et negative potential windows evaluated using Eq. 7 below^56^:

7 $${{C}}_{{s}}=\frac{{{I}}_{{S}}\Delta {t}}{\Delta {V}}$$

where *I*_*s*_ (A g^−1^) represents the specific current, *∆t* (s) is the discharge time from the slope of the discharge part of the GCD plot, and *∆V* (V) is the potential window.

The specific capacitance values for all the sample electrodes were calculated at a specific current of 1 A g^−1^. All APSW-Y4 sample electrodes demonstrated the best electrochemical performance in terms of the calculated *C*_S_ compared to other ratios (1:1, 1:2 and 1:6). The APSW-KOH4 electrodes exhibited the highest specific capacitance of 242.3 F g^−1^, which is in agreement with the high current response from the CV plots.

This is linked to the high SSA and pore volume with a wider pore sizes distribution reported from the textural analysis in which the KOH-activated sample had over an order of magnitude higher amount of pores as compared to the other PEAs (see Fig. 1(d–f)).

In addition, the D/G and D2/G ratio in relation to the smaller effective crystallite size from the Raman analysis also contributes to the enhanced electrochemical performance recorded.

Electrochemical impedance spectroscopy (EIS) study further investigates the capacitive behaviour of the as-fabricated sample electrodes and determines the nature of the electrode in relation to the electrolyte and electrode/electrolyte interface. Figure 5(f) displays the Nyquist plot for the APSW-Y4 sample electrodes with a quasi-vertical line parallel to imaginary Z″-axis in the low frequency region. This is a clear indication that all the samples exhibit mainly a capacitive form of energy storage. The deviation from the complete vertical line in an ideal capacitor is related to the presence of a little ionic resistance even with the dominant capacitive response. The overall length of the Nyquist plot is linked to the nature of the ion diffusion length within the electrode material.

The real Z′-axis intercept in the high frequency region describes the equivalent series resistance (ESR) values for the different sample electrodes and is shown in the inset to Fig. 5(f). This resistance is composed of the resistance of the electrolyte ions, internal resistance of the activated carbon electrode material and the interfacial contact resistance between the current collector and active material. The APSW-KOH4 sample electrodes exhibited the lowest ESR and the shortest diffusion path length in addition to a much more ideal response with respect to its vertical profile parallel to the −Z″-axis. This shows a good electrode–electrolyte interaction and fast ion diffusion occurring at the electrode-electrolyte interface, resulting in low impedance values. The surface interaction at the electrode-electrolyte interface is greatly influenced by the surface chemistry of the electrode material. The presence of a higher D/G defect ratio and the larger amount of amorphous carbon domains in APSW-KOH4 caused higher affinity for aqueous KNO_3_ electrolyte ions resulting in surface hydrophilicity and good carbon surface wettability. Secondly, the large pore volume and increased specific surface area of the APSW-KOH4 allowed for easier accessibility of the electrolyte ions to the electrode surface. Overall, the good textural properties and the distinct interfacial surface features of APSW-KOH4 contributed to low impedance values. It is evident that the APSW-KOH4 electrode displayed the best storage performance due to the high SSA coupled with the combination of a wide distribution of pore sizes, which provides adequate electrochemically active sites and easy diffusion path for the electrolyte ions.

Based on the most promising material and electrochemical characteristics exhibited by the APSW-KOH4 sample electrode, a symmetric device (full cell) with APSW-KOH4//APSW-KOH4 material electrodes was assembled accordingly.

**Figure 6:**
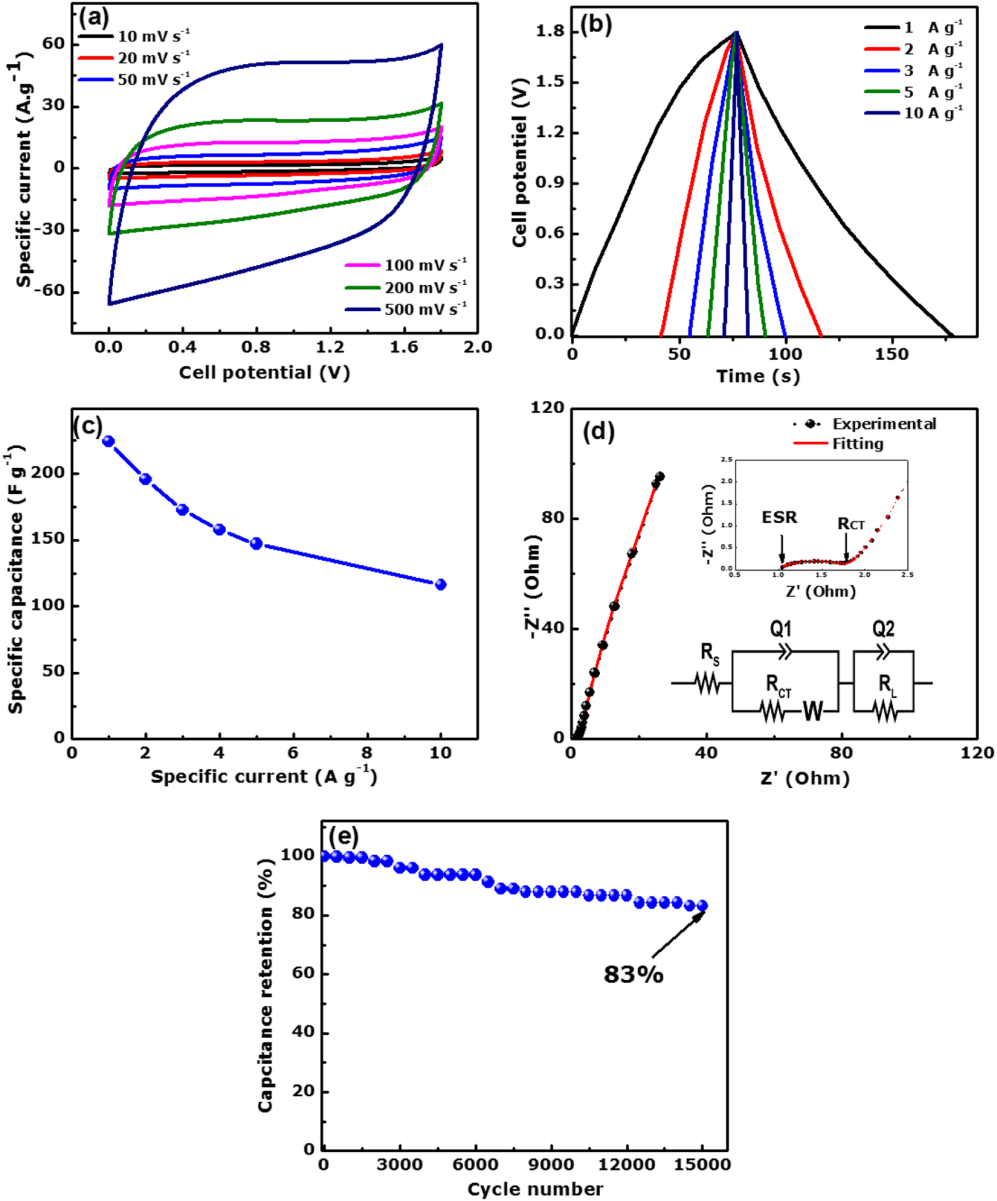
Electrochemical measurement of the APSW-KOH4//APSW-KOH4 symmetric cell displaying. (**a**) CV curves at different scan rates. (**b**) GCD plots at different specific currents, (**c**) specific capacitance as a function of specific current, (**d**) Nyquist plot and (**e**) capacitance retention against cycle number at 5 A g^−1^.

Figure 6(a) presents the CV profiles of the APSW-KOH4//APSW-KOH4 symmetric cell in a similar 2.5 M KNO_3_ neutral electrolyte.

An extended operating cell potential of 1.8 V was obtainable at different scan rates ranging from 10 to 500 mV s^−1^. A typical rectangular shape was retained up to 0.5 V s^−1^ demonstrating its high rate capability and the fast ionic diffusion within the material electrode.

The galvanostatic charge-discharge plots at different specific current (1–10 A g^−1^) is shown in Fig. 6(b). The GCD curves exhibited a symmetric and quasi-linear charge-discharge profile coupled with a small IR drop indicating an impressive electric double layer capacitive feature.

In order to determine the specific capacitance per single electrode C_el_ for the full device, the Eq. 8 below is used^57^;

8 $${{C}}_{{el}}=4\frac{{I}\Delta {t}}{\Delta {V}}$$

where *I* (A g^−1^) is the constant specific current, ∆t (s) is the discharge time from the slop of GCD, and ∆*V* (V) is the full cell potential.

The calculated specific capacitance values (from the GCD curves) as a function of the specific current is shown in Fig. 6(c). A high specific capacitance per electrode of 224.3 F g^−1^ was recorded at 1 A g^−1^ specific current for the symmetric device. This is comparable to the values obtained in the literature on similar activated carbon from other biomass resources^36,58,59^.

The symmetric cell exhibited an equivalent series resistance (ESR) and charge transfer resistance (*R*_CT_) of 1.0 Ω and 0.8 Ω respectively (Fig. 6(d)). The skewed vertical line at the low-frequency region is attributed to some resistance to fast ion-diffusion, which results in its deviation from the ideal capacitive trend.

The equivalent circuit modelled using the data from the Nyquist plot with a ZFIT fitting tool from the EC-Lab software is shown in inset to the Fig. 6(d).

In the circuit, the ESR component is connected in series with the *R*_CT_ and the Warburg impedance (*W*) which are linked in parallel with the constant phase element (Q1).

The constant phase element, CPE (denoted as *Q*) is related to the capacitive behaviour and roughness of the porous activated carbon electrode. This is expressed as:

9 $${Q}=\frac{1}{{T}{({j}\omega )}^{{n}}}$$

where *T* represent the frequency independent constant with dimensions of (F cm^−2^)^*n*^ and *ω* is the angular frequency. The values for *n* range from −1 to 1. When *n* = 0, the CPE element is an ideal resistor; when *n* = 0.5, the CPE transforms to a Warburg element; for *n* = 1, a pure capacitance is depicted; when *n* = −1, the CPE functions as a an inductor^60,61^. A Warburg element is observed at the high to low frequency transition and can be determined by:

10 $${W}=\frac{{A}}{\omega {{j}}^{0.5}}$$

where *A* represents the Warburg coefficient, *ω* is the angular frequency and *n* = 0.5^60,62^.

The skewed vertical line at the low-frequency region from the ideal capacitive trend could be attributed to the leakage resistance (denoted *R*_L_) which is in parallel with the leakage capacitance (*Q*2). The summarized values of the fitting parameters are shown in the Table S4.

Figure 6(e) exhibits the capacity retention of the APSW-KOH4//APSW-KOH4 symmetric device as a function of the cycle number at a specific current 5 A g^−1^. A satisfactory electrochemical stability (83% capacity retention) for the device was recorded even up to 15,000 constant galvanostatic charge-discharge cycles.

**Figure 7:**
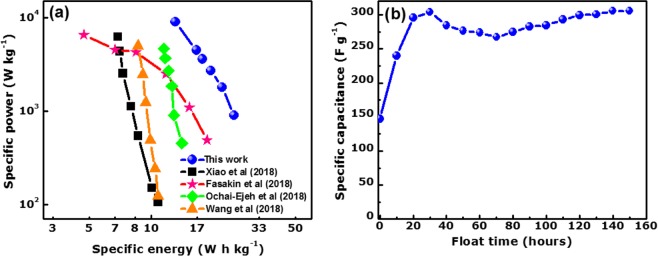
(**a**) Ragone plot of the APSW-KOH4//APSW-KOH4 device and (**b**) specific capacitance against voltage holding time for a period of 150 h at 5 A g^−1^.

The Ragone plot presents the variation of the specific energy (*E*_d_) with the specific power (*P*_d_) as shown in Fig. 7(a). The energy *E*_d_ (Wh kg^−1^) and the specific power (W kg^−1^) for the symmetric cell were evaluated using Eqs 11 and 12 below respectively^57^:

11 $${{E}}_{{d}}=\frac{{{C}}_{{el}}{(\Delta {V})}^{2}}{28.8}$$

12 $${{P}}_{{d}}=3600\frac{{{E}}_{{d}}}{\Delta {t}}$$

where *C*_*el*_ (F g^−1^), ∆*V* (V) and Δ*t* are as described in Eq. (8).

A specific energy of the symmetric cell of 25.2 Wh kg^−1^ was obtained with a corresponding specific power of 0.9 kW kg^−1^ at a specific current 1 A g^−1^.

More importantly, the specific energy was still maintained at 13.0 Wh kg^−1^ with a specific power of 9 kW kg^−1^ even with the specific current being increased to 10 times its original value (10 A g^−1^).

The reported values are much higher as compared to other carbonaceous raw peanut shell activated with K_2_CO_3_ and KHCO_3_ as shown in Fig. S7 of the supporting information.

A comparison of the results from this study with other similar selected reports in the literature on activated carbon from biomass waste materials is presented in Fig. 7(a) and Table 1.

**Table 1:** Comparison of electrochemical results of activated carbon from various biomass.

Materials	PEA	Electrolyte	Specific current (A g^−1^)	Specific energy (Wh kg^−1^)	Specific power (W kg^−1^)	Ref
Peanut shell	ZnCl_2_	1 M H_2_SO_4_	0.5	10.8	106.9	^27^
Banana peels	KOH	1 M NaNO_3_	0.5	18.6	485	^36^
Soybean Pods	KOH	1 M Na_2_SO_4_	0.5	22.28	450	^63^
Quercus Suber	KHCO_3_	3 M KNO_3_	0.5	14	450	^35^
Quercus Suber	KOH	1 M Na_2_SO_4_	0.5	18.6	449.4	^64^
Pueraria	K_2_CO_3_	6 M KOH	0.5	8.46	123	^65^
Fresh clover stems	KOH	1 M H_2_SO_4_	0.5	10.7	125	^66^
Coconut shell	KOH	PVA-KOH-CB gel electrolyte	0.5	11	325	^67^
Sawdust	KOH	6 M KOH	0.5	8.4	250	^68^
Waste bagasse	KOH	1 M Na_2_SO_4_	0.5	22.3	220.9	^58^
White Sugar	KOH	1 M Na_2_SO_4_	1	19	750	^69^
Peanut shell waste	**KOH**	**2.5 M KNO**_**3**_	**1**	**25.2**	**900**	**This work**

The specific energy and specific power reported in this work for APSW-KOH4 in 2.5 M KNO_3_ was relatively higher and in some cases, comparable with those related reports on symmetric supercapacitors based on biomass-derived carbons operated in neutral aqueous electrolytes.

For example, F. O. Ochai-Ejeh *et al*.^49^ reported the synthesis of porous carbon from Quercus Suber using KHCO_3_. The full symmetric device was done using the similar aqueous electrolyte 3 M KNO_3_ and obtained a specific energy of 14 Wh kg^−1^ with a specific power of 450 W kg^−1^ at 0.5 A g^−1^ specific current.

Recently, Z. Xiao and co-workers^27^ produced porous carbon using the same biomass peanut shells activated with ZnCl_2_. In their report, the electrochemical measurement of the symmetric cell was tested in 1 M H_2_SO_4_ aqueous electrolyte. The values of the specific energy and specific power were 10.8 Wh kg^−1^ and 106.9 W kg^−1^ respectively at 0.5 A g^−1^.

The high specific energy and power acquired in our work can be related to the high SSA with a large pore volume and the significant degree of amorphous carbon present in the APSW-KOH4 sample contributing to enhancing the electrochemical results.

To further evaluate the stability of the obtained symmetric device, a voltage holding was carried out at an operating cell potential of 1.8 V for a period of 150 h (more than 6 days).

Figure 7(b) displays the specific capacitance as function of the floating time for each 10 h interval. It is worthy to note that the recorded *C*_S_ increase remarkably for the first 30 h following a small drop between the 40 to 80 h periods. Thereafter, another increase in charge storage capability was observed up to the 90 h mark before stabilising for higher floating times. An overall increase in the specific capacitance values was observed from 147 to 307 F g^−1^.

The improvement in the recorded *C*_S_ can be attributed to the accessibility of the ions to initially inaccessible pore sites through increased wettability and a large diffusion of ions between the electrolyte/electrode during the long floating time. A higher specific energy of 34.5 Wh kg^−1^ at 5 A g^−1^ was obtained after 150 h floating time as compared to the initially recorded specific energy of 16.5 Wh kg^−1^ before floating test at the same specific current value.

It is obvious that the increase of *C*_S_ after being subjected to floating tests also generates a simultaneous increase in the device specific energy (twice compared to the initial value).

## Conclusion

The activated carbon from peanut shell waste was successfully synthesized via a two-step carbonization and activation process at elevated temperature using different porosity enhancing agents KHCO_3,_ K_2_CO_3_ and KOH with varying mass ratios (CPSW:PEA). Among the PEAs, the carbonisation/activation process with KOH resulted in a porous carbon material with the best textural properties.

The APSW-KOH4 exhibited the largest specific surface area of 2547 m^2^ g^−1^, and pore volume of 1.2 cm^3^ g^−1^. The complex pore framework composed of cavities facilitated efficient ionic transport within the material. The Raman spectra clearly illustrated the degree of amorphous carbon present in the lattice structure. XPS analysis of the APSW-KOH4 confirmed the presence of a high concentration of carbon with a little amount of nitrogen and oxygen.

The electrochemical measurement performed on material electrodes in half-cell set-ups demonstrated a reversible operating capability in both the positive (0.0–0.9 V) and the negative (−0.9–0.0 V) potential windows. The assembled symmetric device using the best materials from the textural, structural and initial electrochemical tests (APSW-KOH4//APSW-KOH4) displayed a specific capacitance of 224.3 F g^−1^, a high specific energy of 25.2 Wh kg^−1^ corresponding to power density of 0.9 kW kg^−1^ at 1 A g^−1^ in a cell potential of 1.8 V.

In addition, a good device stability was demonstrated with 83% capacity retention after 15000 cycles and an improvement in electrochemical energy storage capability over a 150 hours floating time (more than 6 days).

The obtained results validate the potential adoption of the porous activated carbon from peanut shell waste as a suitable electrode material for enhanced energy storage.

## Supplementary information

Effect of porosity enhancing agents on the electrochemical performance of high-energy ultracapacitor electrodes derived from peanut shell waste
