# Supplementary material for: Effect of porosity enhancing agents on the electrochemical performance of high-energy ultracapacitor electrodes derived from peanut shell waste
Source: Sci Rep. 2019 Sep 20;9:13673. doi: 10.1038/s41598-019-50189-x (PMC6754434; doi:10.1038/s41598-019-50189-x)
Supplement: Supplementary file 1 — Effect of porosity enhancing agents on the electrochemical performance of high-energy ultracapacitor electrodes derived from peanut shell waste [file 41598_2019_50189_MOESM1_ESM.docx]

**Effect of porosity enhancing agents on the electrochemical performance of high-energy ultracapacitor electrodes derived from peanut shell waste**

**N. F. Sylla ^a^, N. M. Ndiaye ^a^, B. D. Ngom ^b^,** **D. Momodu**

**M. J. Madito, B. K. Mutuma ^a^ and N. Manyala ^a^***

^a^ Department of Physics, Institute of Applied Materials, SARChI Chair in Carbon Technology and Materials, University of Pretoria, Pretoria 0028, South Africa

^b^ Laboratoire d’Energie, de Photonique et de Nano-Fabrication, Faculté des Sciences et Techniques Université Cheikh Anta Diop de Dakar (UCAD) B.P. 25114 Dakar-Fann Dakar, Sénégal

*Corresponding author email: [ncholu.manyala@up.ac.za](mailto:ncholu.manyala@up.ac.za), Tel: + (27)12 420 3549

Fax: + (27)12 420 2516

**Supporting information**

| **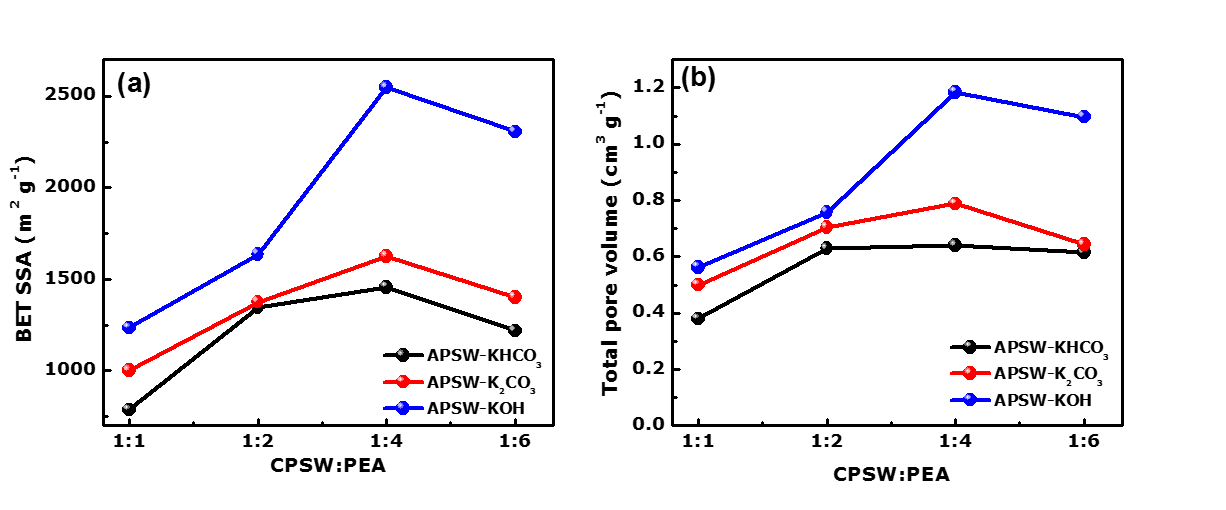** |
| --- |

**Figure S1**: (a) The plot of specific surface area and (b) Total pore volume as a function of the APSW at different activated agent for different ratios.

| 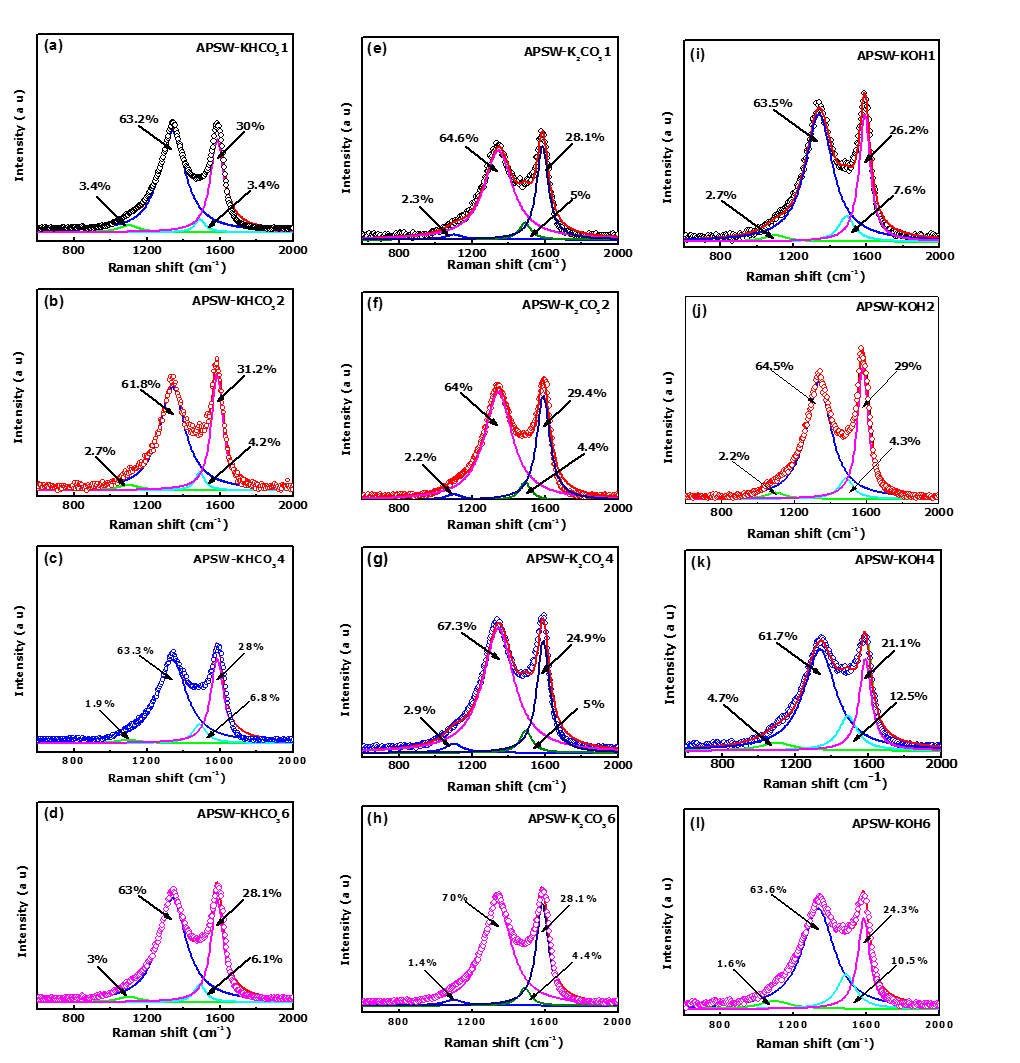 |
| --- |

**Figure S2**. Deconvolution of the Raman spectra showing the integral areas of the D1, D, D2 and G peaks for all the APSW-Yx samples.

**Table S1**: Specific surface area (SSA) data of different APSW-Yx samples

| **CPSW:PEA** | **SSA (m^2^ g^-1^)** | | |
| --- | --- | --- | --- |
|  | KHCO_3_ | K_2_CO_3_ | KOH |
| **1:1** | 787 | 1002 | 1235 |
| **1:2** | 1348 | 1376 | 1637 |
| **1:4** | 1457 | 1625 | 2547 |
| **1:6** | 1219 | 1401 | 2306 |

**Table S2**: D/G ratio, effective crystallite size L_a_ and D peak FWHM data of different APSW-Yx samples

| **CPSW:PEA** | **D/G** | | | **L_a_ (nm) = 4,96/(D/G)*** | | | **D peak FWHM (cm^-1^)** | | |
| --- | --- | --- | --- | --- | --- | --- | --- | --- | --- |
|  | KHCO_3_ | K_2_CO_3_ | KOH | KHCO_3_ | K_2_CO_3_ | KOH | KHCO_3_ | K_2_CO_3_ | KOH |
| **1:1** | 2.1 | 2.3 | 2.4 | 2.4 | 2.2 | 2.1 | 170.3 | 183.4 | 192.7 |
| **1:2** | 2.0 | 2.2 | 2.2 | 2.5 | 2.3 | 2.3 | 160.1 | 169.3 | 187.1 |
| **1:4** | 2.3 | 2.7 | 2.9 | 2.2 | 1.8 | 1.7 | 194.6 | 206.4 | 225.7 |
| **1:6** | 2.3 | 2.7 | 2.6 | 2.2 | 1.8 | 1.9 | 187.2 | 201.9 | 215.7 |

^*^ Equation adopted from ^1^

**Table S3**: Atomic concentration data of the APSW-KHCO_3_, APSW-K_2_CO_3_ and APSW-KOH at a mass ratio of 1 to 4 samples

|  | Atomic Conc (%) | | |
| --- | --- | --- | --- |
|  | C1S | O1S | N1S |
| APSW-KHCO_3_4 | 85.2 | 14.2 | 0.6 |
| APSW-K_2_CO_3_4 | 86.9 | 11.9 | 1.2 |
| APSW-KOH4 | 87.9 | 11.5 | 0.6 |

|  |
| --- |

**Figure S3**. (a) D peak FWHM and (b) effective crystallite size L_a_ as function of APSW-KHCO_3_, APSW-K_2_CO_3_, APSW-KOH at different mass ratios.

| 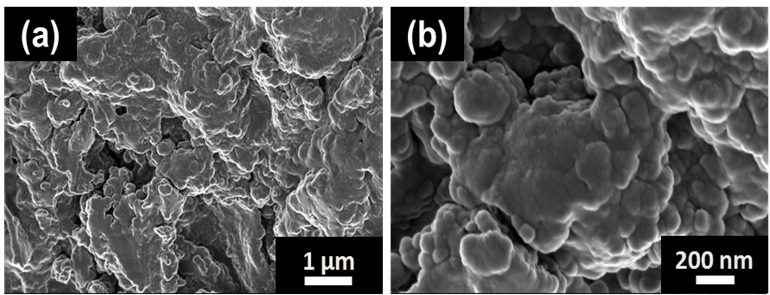 |
| --- |

**Figure S4.** SEM images of the raw PSW at: (a) low and (b) high magnification

| 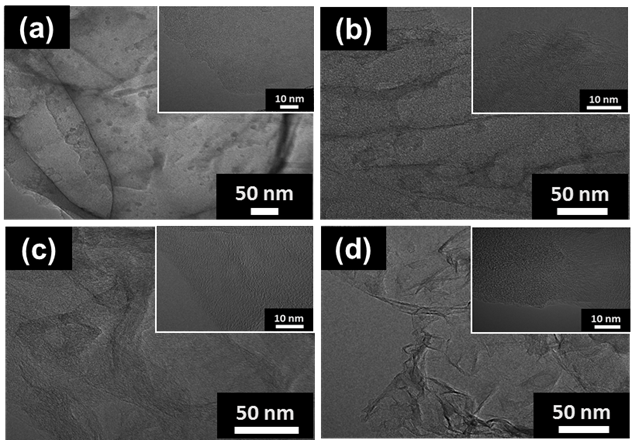 |
| --- |

**Figure S5.** HRTEM images at low and high magnification (inset) of (a) CPSW; (b) APSW-KHCO_3_4; (c) APSW-K_2_CO_3_4 and (d) APSW-KOH4

| **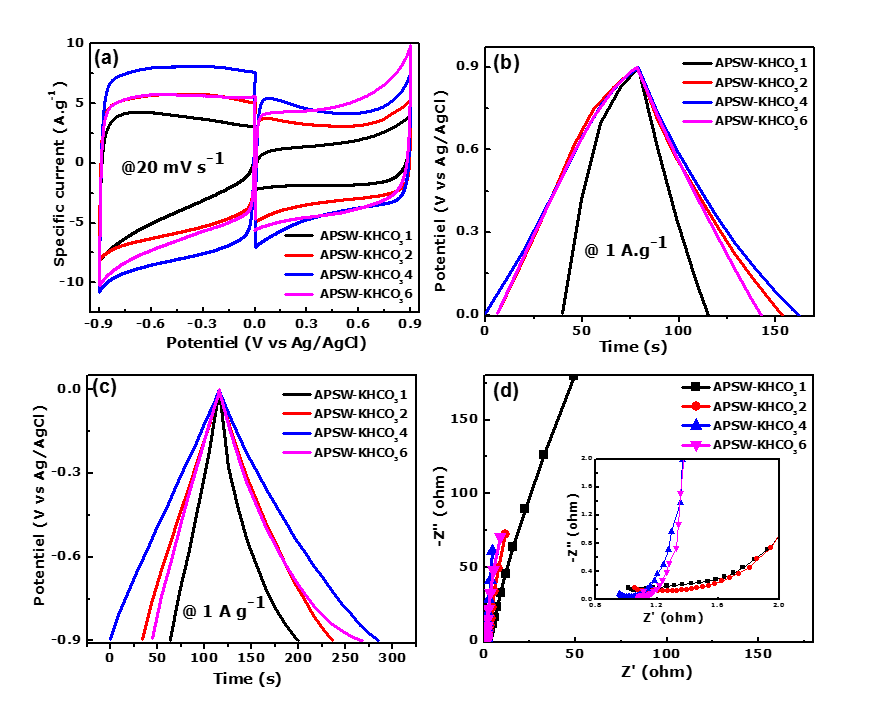** |
| --- |

**Figure S6.** Electrochemical measurement of the APSW-KHCO_3_ electrodes at different concentrations: (a) CV curves at a scan rates 20 mV s^-1^, (b) and (c) GCD plots at a specific current of 1 A g^-1^ in the positive and negative potential windows, respectively and (d) Nyquist plots.

| 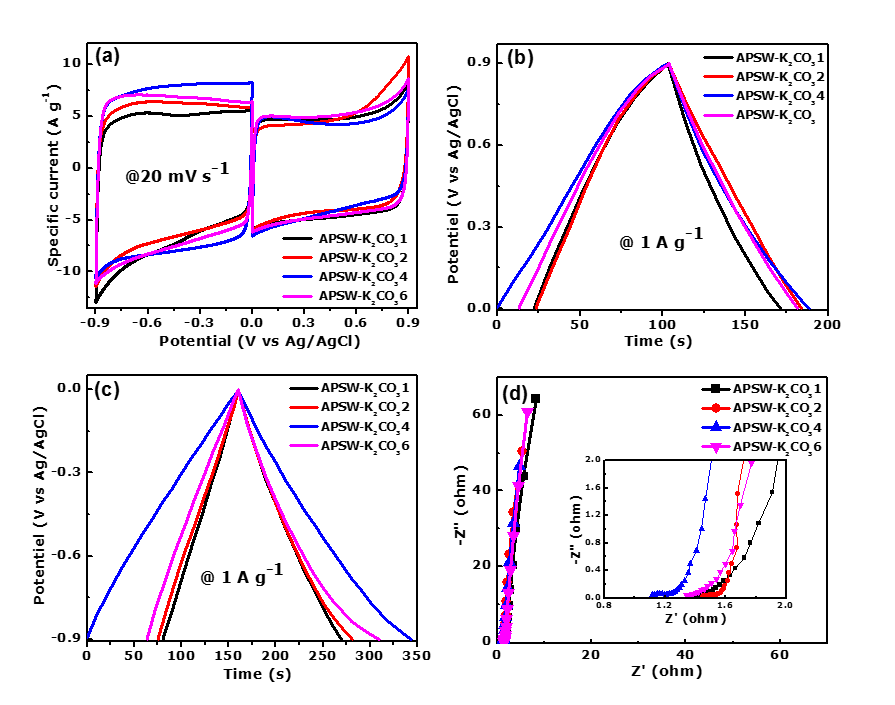 |
| --- |

**Figure S7.** Electrochemical measurement of the APSW-K_2_CO_3_ electrodes at different concentrations: (a) CV curves at a scan rates 20 mV s^-1^, (b) and (c) GCD plots at a specific current of 1 A g^-1^ in the positive and negative potential windows, respectively (d) Nyquist plots.

| **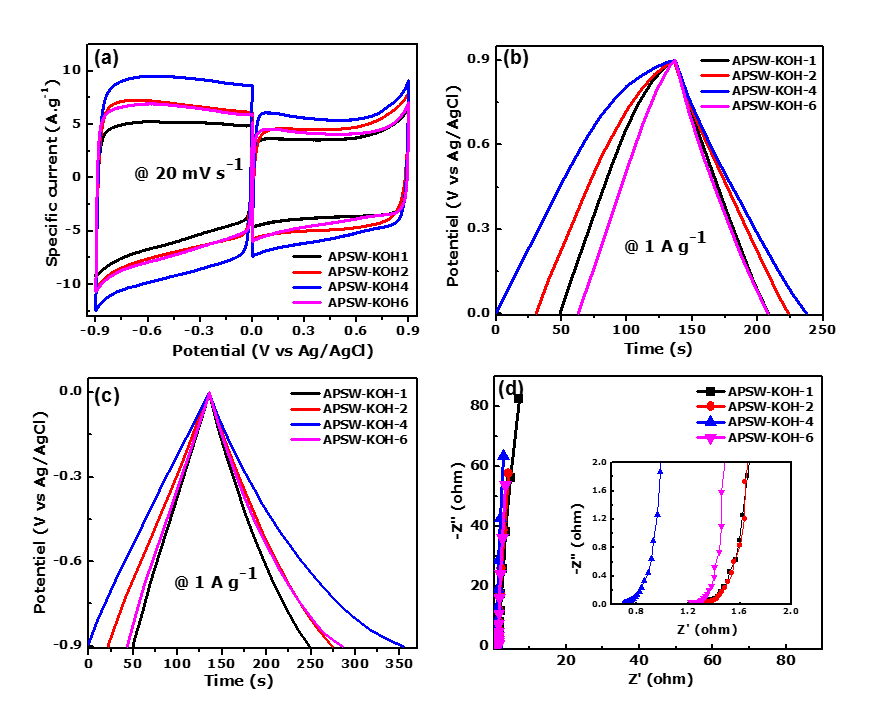** |
| --- |

**Figure S8.** Electrochemical measurement of the APSW-KOH electrodes at different concentrations: (a) CV curves at a scan rates 20 mV s^-1^, (b) and (c) GCD plots at a specific current of 1 A g^-1^ in the positive and negative potential windows, respectively (d) Nyquist plots.

**Table S4.** EIS fitting parameters obtained from the complex non-linear least square (CNLS) method of the equivalent circuit shown in the inset to Fig. 6d

| **Electrode** | ***R*_s_ (Ω)** | ***R*_CT_ (Ω)** | ***Q2* (F)** | ***R*_L_ (Ω)** |
| --- | --- | --- | --- | --- |
| **APSW-KOH4 //APSW-KOH4** | 1.01 | 0.79 | 0.146 | 7.99 × 10^2^ |
| X/$\sqrt{N}$= 0.366, *Q2* ≡ *leakage capacitance*_,_ | | | | |

| **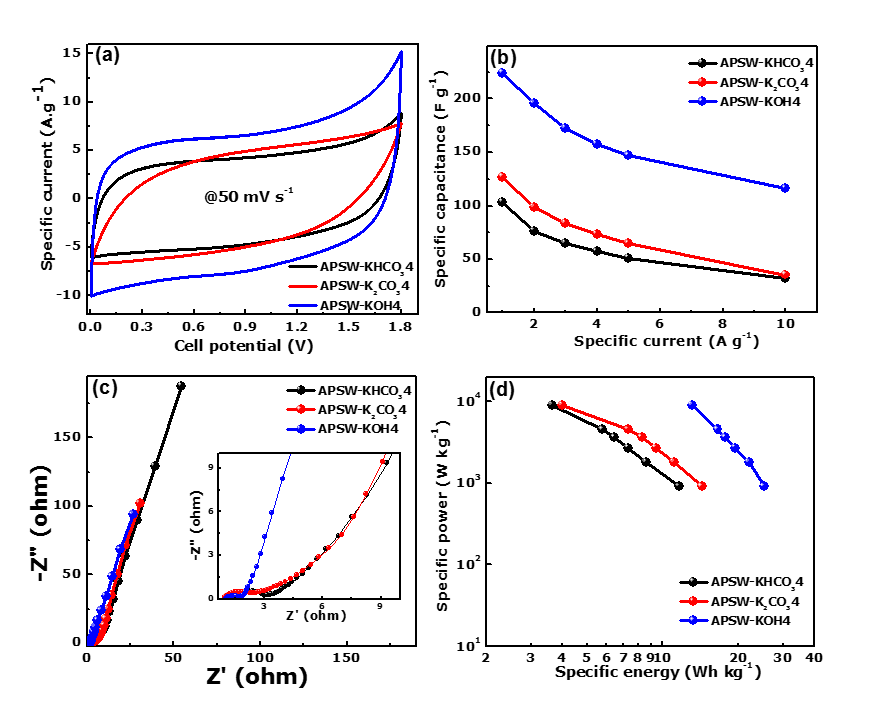** |
| --- |

**Figure S9.** Comparison of the symmetric electrodes APSW-KHCO_3_//APSWKHCO_3_, APSWK_2_CO_3_//APSWK_2_CO_3_, APSW-KOH//APSW-KOH at a mass ratio 1:4: (a) CV curves, (b) specific capacitance as function of the specific current, (c) Nyquist plots and (d) Comparison of the Ragone plot for the three symmetric devices

**References**

1. Endo, M. & Pimenta, M. A. Origin of dispersive effects of the raman d band in carbon materials. *Phys. Rev. B - Condens. Matter Mater. Phys.* **59,** R6585–R6588 (1999).
